# Experimental pharmacology in precision medicine

**DOI:** 10.1002/prp2.1147

**Published:** 2023-10-26

**Authors:** Alicja Urbaniak, Kenneth E. Thummel, Ayoade N. Alade, Allan E. Rettie, Bhagwat Prasad, Amedeo De Nicolò, Jennifer H. Martin, David N. Sheppard, Michael F. Jarvis

**Affiliations:** ^1^ Department of Biochemistry and Molecular Biology University of Arkansas for Medical Sciences Little Rock Arkansas USA; ^2^ School of Pharmacy University of Washington Seattle Washington USA; ^3^ Department of Pharmaceutical Sciences Washington State University Spokane Washington USA; ^4^ University of Turin Turin Italy; ^5^ The University of Newcastle Hunter Medical Research Institute New Lambton New South Wales Australia; ^6^ School of Physiology, Pharmacology and Neuroscience University of Bristol Bristol UK; ^7^ Pharmaceutical Sciences University of Illinois‐Chicago Chicago Illinois USA

**Abbreviations**
5FU: 5‐fluorouracil
ADME: absorption, distribution, metabolism and elimination
ATV/r: ritonavir‐boosted atazanavir
cART: combined antiretroviral therapy
CF: cystic fibrosis
CFTR: cystic fibrosis transmembrane conductance regulator
DDI: drug‐drug interaction
DTG: dolutegravir
F‐CAP: functional annotation of all coding variation
GMR: geometric‐mean ratio
HLA: human leukocyte antigen
INR: international normalized ratio
LC‐MS/MS: liquid chromatography with tandem mass spectrometry
NRTI: nucleoside retro‐transcriptase inhibitor
PBMC: peripheral blood mononuclear cell
PBPK: physiologically based pharmacokinetic
RIF: rifampicin
TDM: therapeutic drug monitoring

## 1 INTRODUCTION

Following the initial sequencing of the human genome, it was widely predicted that this knowledge would lead to transformational advances in the identification, prevention, and treatment of disease.^1^ The terms “Precision Medicine” and “Personalized Medicine” have been used interchangeably over the last two decades to describe almost all applications of genomic information in the development and use of medicinal interventions.^2^ While there are notable clinical advances that exemplify the highly effective benefit/risk profiles of precision medicine‐driven approaches^1^, ^2^ the general concept of targeting specific interventions to individual patients continues to be a rich area of translational research.

The 19th World Congress of Basic & Clinical Pharmacology (WCP2023) was held in Glasgow Scotland from July 2 to 7, 2023. The British Pharmacological Society and International Union of Basic and Clinical Pharmacologists (IUPHAR) hosted this important international meeting. WCP2023 was attended by more than 2000 delegates from over 80 countries. It is noteworthy that many of the WCP2023 scientific sessions, across a wide range of therapeutic areas, addressed ongoing research relevant to the current state and aspirational goals of precision medicine approaches for improving human health.

During WCP2023, the symposium “Experimental Pharmacology in Precision Medicine” presented recent advances and current challenges in the application of precision medicine to diverse areas, including drug metabolism, drug–drug interactions, therapeutic drug dose monitoring and therapeutic interventions for cystic fibrosis (CF). The speakers were Drs. K.E. Thummel, B. Prasad, J. Martin, and D.N. Sheppard and the co‐chairs of the symposium were Drs. A. Urbaniak and M.F. Jarvis. Consistent with the goal of highlighting research from early career investigators, the WCP2023 organizers also invited Dr. A. De Nicolò to give an oral presentation based on his submitted abstract on the metabolism of antiretroviral drugs. In the following sections of this article, each of the speakers provides a summary of their oral presentations from this symposium.

### 1.1 Implementing precision medicine: Overview of progress. (K.E. Thummel)

While definitions vary, Precision Medicine is generally described as a medical model that utilizes molecular information (e.g., “omic” technologies) to improve the precision with which patients are stratified to better inform clinical decisions, most notably drug selection, and dosing. Significant advances in this medical domain have occurred over the past 25 years, including testing for pharmacogene variations that affect drug safety and efficacy. One approach to categorizing pharmacogenes proposed by Bill Evans and Mary Relling,^3^ groups them into four categories: those affecting host susceptibility to adverse response, those implicated in disease pathogenesis, those affecting drug receptor function, and those affecting drug disposition. Perhaps the greatest strides towards broad clinical implementation have come with testing for variations in genes that encode human leukocyte antigen (HLA) proteins that affect the risk of life‐threatening adverse responses to abacavir, carbamazepine, and other drugs. For example, results of a recent multi‐center study from Mounzer et al.,^4^ showed a steady, marked reduction in abacavir hypersensitivity reaction incidence between 2007 and 2015 that accompanied increased implementation of HLA‐B*5701 testing during the same period. The high negative predictive value of the test likely ensured near‐complete adoption of the test before abacavir administration in the treatment of HIV. Similar clinical utility and wide‐spread adoption have been demonstrated for a rapidly growing list of “companion diagnostic” tests to identify specific tumor variations that predict drug‐specific anti‐cancer efficacy, as well as testing for mutations in the cystic fibrosis transmembrane conductance regulator (*CFTR*) gene that inform drug selection and efficacy in the treatment of CF.^5^, ^6^

Hundreds of drug‐gene pairs have been identified as potential candidates for Precision Medicine, and many have been proposed for implementation by experts in the field^7^ and organizations such as the Clinical Pharmacogenetics Implementation Consortium (CPIC), often with the provision of levels of evidence demonstrating the clinical value of genetic testing and detailed decision trees for interpreting testing results (cpicpgx.org). The FDA also maintains the Pharmacogenetic Biomarker Bulletin which details relevant pharmacogenetic information found on drug labels to inform both clinical and research communities (FDA.gov). Interestingly, with respect to host gene variation that affects drug disposition or response, most drug labels describe how genetic testing results could be used, if results are available, but do not mandate preemptive testing before drug use. A limitation of preemptive testing often cited is the fact that a majority of the variation in drug disposition or response is not explained by known gene variation. This is true even for the narrow therapeutic index drug warfarin, with a pharmacological response that is affected by variation in *CYP2C9* and *VKORC1* genes. Nonetheless, based on evidence of clinical validity and utility of precision testing, some healthcare centers such as Vanderbilt have adopted preemptive testing for variation in these and other pharmacogenes (PREDICT) to inform drug therapy decisions over the life of a patient, starting with an index drug–gene pair, potentially enhancing clinical utility and cost‐effectiveness.^8^ Although any one drug‐gene pair test may have relatively low positive or negative predictive value, testing for multiple gene‐drug pairs preemptively may bring more tangible benefits in the long run. To evaluate the underlying premise of this Precision testing model, the European UPgx consortium conducted a multi‐center, cluster‐randomized, crossover trial (PREPARE) that involved preemptive testing for variation (≥1% minor allele frequency) in 12 well‐established pharmacogenes and association with patient‐reported adverse drug reactions. Investigators reported a 30% reduction in the incidence of adverse drug reactions with preemptive pharmacogenetic testing, compared to control.^9^

Looking forward, the utility of Precision Medicine might be improved through an expansion of testing that currently focuses on candidate genes and a limited repertoire of common variants. Testing for rare variation, local epistasis (haplotypes), epigenetics, and polygenic traits have all been touted as additional biomarkers of inter‐individual differences in drug response. Implementation of whole genome or exome sequencing as a testing platform opens the door for the identification of rare nonsense or missense variation, which is pervasive in pharmacogenes.^10^ Of course, it then becomes necessary to assess by some means the functional impact of variants that are unlikely to be the sole focus of a targeted pharmacogenetic study. To address this problem, Fowler and colleagues^11^ proposed F‐CAP (Functional Annotation of All Coding Variation) which is enabled by deep mutational scanning coupled to a phenotype reporter such as protein stability, enzyme catalytic activity, or receptor‐ligand interactions. Recent successes with TPMT, VKOR, CYP2C9, and NUDT15 highlight its potential value as part of the Precision Medicine tool chest. Indeed, evaluation of the functional effects of all possible amino acid substitutions in NUDT1*5* nicely illustrated how the approach can separate the valuable “wheat” from the “chaff” among all rare variants seen in the human population.^12^

In addition to the characterization of rare variants in the *VKORC1* and *CYP2C9* genes, we recently examined, using a well‐characterized, relatively large (*n* = 350) human liver bank, the contribution of genotypic and phenotypic differences in the *CYP4F2/CYP4F11* genes and associated enzyme products to variability in vitamin K catabolism (*unpublished*), which can also affect warfarin anti‐coagulant response.^13^ The *CYP4F* locus showed extensive linkage disequilibrium between the 13 common single nucleotide variants identified (including 5 missense), resulting in 20 haplotypes, 7 of which had a frequency greater than 5%. Microsomal CYP4F2 and CYP4F11 protein abundances and phylloquinone ω‐hydroxylation kinetics (K_m_, V_max_, CL_int_) were measured and tested for association with single missense single nucleotide variant genotypes and haplotype derived diplotypes. Results revealed that most haplotypes were not associated with altered metabolic kinetics and that the single CYP4F2*3 variant (Val433Met) was just as predictive of reduced protein abundance (73% lower than reference in the homozygous variant group) and CL_int_ as the two haplotypes that contained the variant. Interestingly, the results also revealed extensive inter‐liver variability in the CL_int_ among all other livers that did not contain the CYP4F2*3 allele and that could not be explained by other gene variants. In addition, although the abundance of CYP4F11 protein was much lower than that of CYP4F2 in those “reference” livers, it exceeded CYP4F2 abundance in some of the livers with the homozygous CYP4F2*3 genotype. Thus, the largely unexplained variation in phylloquinone CL_int_ might contribute significantly to interindividual differences in warfarin anti‐coagulant response. Collection of so‐called “liquid biopsies” of the liver^14^ and quantitative measurements of CYP4F2 and CYP4F11 protein abundances in liver‐derived exosomes might improve predictions of the warfarin dose needed to achieve a therapeutic international normalized ratio (INR), well beyond what genetic testing alone provides. Moreover, this may be a generalizable biomarker approach for other drug‐gene pairs. In summary, with input from multiple stakeholders, the application of Precision Medicine to inform pharmacotherapy is likely to undergo further refinement with an expansion of molecular tools, databases, and decision support systems available to the healthcare practice community, hopefully leading to improvements in drug safety and efficacy.

### 1.2 Quantitative proteomics in translational pharmacology. (B. Prasad)

Quantitative proteomics has emerged as an important technique in translating in vitro or animal data on drug absorption, distribution, metabolism, and elimination (ADME) to clinics as well as predicting interindividual variability in these processes. Quantitative proteomics applies to three key areas of translational pharmacology. First, the technique can characterize cross‐species differences in the levels of drug‐metabolizing enzymes and transporters. For example, the abundance of organic anion and cation transporters in the kidney is substantially higher in mice and rats as compared to humans.^15^ These proteomic differences explain species‐dependent renal clearance and drug‐induced kidney injury. Thus, quantitative proteomics is crucial for making informed decisions with respect to the interpretation of preclinical pharmacokinetics and toxicity data. Second, in vitro to in vivo extrapolation of drug metabolism and transport can be accomplished by integrating quantitative proteomics data with in vitro results. For example, the dose‐limiting gastrointestinal toxicity of intravenous irinotecan can be explained by the preferential formation and accumulation of the toxic metabolite, SN‐38 in enterocytes. This is due to the high expression of carboxylesterase 2 and low expression of UDP‐glucuronosyltransferase 1A1, the enzymes responsible for the formation and elimination of SN‐38, respectively, in enterocytes as compared to hepatocytes.^16^ Finally, quantitative proteomics data can be used for the characterization of interindividual variability in drug disposition and response. Clinical trials for drug safety and efficacy rarely account for high‐interindividual variability in drug ADME, thus posing a risk of unpredictable drug safety concerns including drug–drug interactions (DDIs) in populations underrepresented in clinical trials, e.g., children, elderly, certain races, pregnant women, etc. Current data on population variability are sparse and do not address technical variability. Furthermore, while significant progress has been made to utilize in vitro models to predict drug ADME using physiologically based pharmacokinetic (PBPK) models, these models require comprehensive physiological data on inter‐individual variability. In particular, PBPK models require quantitative information on the levels and activity of individual pathways involved in drug disposition across different tissues and populations (healthy vs. diseased or children vs. adults). Quantitative proteomics revealed that UGT2B17 is one of the most highly variable intestinal enzymes that is rarely expressed in children below 9 years and the enzyme expression is ~3‐fold lower in women than men.^17^ UGT2B17 gene deletion carriers and women are vulnerable to slower metabolism and increased bioavailability of diclofenac. Considering the cardiotoxicity risk of diclofenac, proteomics‐informed PBPK modeling suggests that the diclofenac dose should be decreased in women and the carriers of UGT2B17 gene deletion.^18^ Similarly, quantitative proteomics is useful in the discovery and quantification of efficacy biomarkers. For example, warfarin is a narrow therapeutic index drug that requires stratification of subjects based on the levels of descarboxylated prothrombin. Quantitative proteomics can simultaneously quantify both carboxylated and descarboxylated forms of prothrombin, thus useful in stratifying patients for precision warfarin therapy.^19^

### 1.3 Plasma and intracellular concentrations of ritonavir, atazanavir, and dolutegravir in the presence of rifampicin in the context of a dose escalation study. (A. De Nicolò)

Ritonavir‐boosted atazanavir (ATV/r) is an important therapeutic option for combined antiretroviral therapy (cART). Nevertheless, these drugs are prone to DDIs with cytochrome inducers, such as rifampicin (RIF). Therefore, a dose escalation study was planned to assess the pharmacokinetic effectiveness and safety of administering ATV/r twice‐daily (bid) instead of once daily (qd) to overcome RIF inducing effect. For this purpose, healthy volunteers living with HIV with suppressed viral load and who are already on ATV/r containing regimens were enrolled. The trial consisted of sequential periods: in the first period (PK1, 1 week) participants were held on their previous ATV/r 300/100 mg qd, with a nucleoside retro‐transcriptase inhibitor (NRTI) backbone; in the second period (PK2, 2 weeks long) standard RIF 600 mg qd dose and dolutegravir (DTG, a potent integrase inhibitor, as a safety drug) 50 mg bid were added; in the third period (PK3, 1 week) ATV/r increased to 300/100 mg bid and, finally, in the fourth period (PK4, 1 week) RIF dose was doubled to the maximum of 1200 mg qd. Then, RIF was withdrawn. At the end of each period, from PK1 to PK4, ATV, RTV, and DTG were quantified in plasma and peripheral blood mononuclear cells (PBMCs) by an LC–MS/MS validated method, to evaluate steady‐state trough concentrations.

Plasma concentrations of ATV/r dropped dramatically after the addition of RIF (ATV geometric‐mean ratio GMR, [all compared to PK1] 0.034, CI_90%_ 0.026–0.045, *p* = .021), but dose escalation did compensate adequately for the inductive effect of RIF (ATV GMR at PK3 vs PK1, 1.04, CI_90%_ 0.598–1.830). No Grade 3 or 4 safety events were observed.

Furthermore, a larger preliminary analysis of intra‐PBMC concentrations from the first 15 participants confirmed the drop in ATV/r concentrations (ATV GMR 0.14, CI_90%_ 0.09–0.27) in the presence of RIF, and the effective compensation provided by dose escalation (ATV GMR 1.14 CI_90%_ 0.74–1.76). Further increasing the RIF dose did not show any significant impact (ATV GMR PK4/PK3 0.93, CI_90%_ 0.71–1.22). DTG appeared to significantly increase after ATV/r dose escalation (GMR PK3/PK2 1.37, CI_90%_ 1.37–3.53) but RIF 1200 mg restored the original concentrations (GMR PK4/PK2 1.53, CI_90%_ 0.98–2.40).

These preliminary data are extremely encouraging and, if definitively confirmed, would suggest the effectiveness and safety of increasing ATV/r dose to overcome the effect of RIF, even at its highest dose of 1200 mg, which is being tested for shortening the treatment of tuberculosis or in cases of complicated tuberculosis infection.

### 1.4 Precision medicine in practice—Do not forget the pharmacology! (J. H. Martin)

Precision Medicine is a nebulous term, with scientific definitions including genetic code mutations, the expression of which can be altered by the use of a specifically developed therapeutic, the development of new molecules to bind a specific ‘target’ or cell, ligand of interest, a serendipitous ‘finding’ based on mathematical and chemical profiling, or use of existing drugs knowledge of which suggests they may bind a new target of interest.^20^, ^21^ Clinicians, however, tend to understand precision medicine as the following: right decision (treat or not, use therapeutic or non‐therapeutic); right therapeutic; right combination; right timing; and right dose for an individual patient. For clinicians, this ‘precision’ process also includes consideration of the clinical trial data for the patient group being treated AND finessing that data to the individual biological and pharmacological variables either evident or likely to be evident, based on knowledge about comorbidity and body size of that patient.

Whatever the definition, it is reasonable to consider the utility of such. For example, has a focus on ‘targets’ to improve *precision* overlooked the well‐known principles of pharmacology, physiology, and cell/disease biology?^21^ Has society received the improved health outcomes expected from the development of medicines that block specific targets? Maybe. Therapies for relatively homogeneous disorders such as blood malignancies, checkpoint inhibitors, and a few others serve as valuable examples. It may be no coincidence that there is little literature measuring a link between funding and clinical improvements from such “targeted drugs”, and even less showing mortality or quality of life benefit^22^, ^23^ with comparatively more clinical benefits arguably being from medicines that more broadly interrupt, or change the speed of a biological pathway, such as drugs that affect the renin‐angiotensin system, statins, steroids, or anti‐rejection drugs. It is noted that clinical benefit from some very targeted therapies was always difficult to ascertain, as the placebo group often crossed to the treatment group. Has this more recent investment into a narrow “genetic” definition of precision medicine neglected important pharmacological aspects such as an individual's own dose–response curve?

An example of exciting precision science without clinical translation is evidenced in the examination of the actual clinical utility of genetic tests for warfarin use. In the 2013 randomized controlled trial,^24^ the average percentage of time in the therapeutic INR range was 67.4% in the genotype‐guided versus 60.3% in the control group, with relatively little clinical significance for an individual patient. Time to reach therapeutic INR was numerically slightly different but with overlapping interquartile ranges, and the median for both groups was 3–4 weeks, often long outside clinical inpatient stay and thus with little benefit to clinical flow or hospital bed stay. Importantly, there were no differences in bleeding between the two groups.

Cancer medicines often have narrow therapeutic windows; toxicity can be severe and sometimes fatal, but inadequate dose intensity reduces efficacy and survival. Determining the optimal dose for each patient is difficult, with the body surface area used most commonly for chemotherapy and flat dosing for tyrosine kinase inhibitors, despite accumulating evidence of a wide range of exposures in individual patients with many receiving a suboptimal dose with these strategies. For clinicians, precision medicine has more recently tended to focus on phenotype testing for most patients. Therapeutic drug monitoring (measuring the drug concentration in a biological fluid, usually plasma) (TDM) is an accepted and well‐validated method to guide dose adjustments for individual patients to improve precision. However, implementing TDM in routine care has been difficult outside a research context. To improve the precision with the first dose, the development of genotyping of various proteins involved in drug elimination and activity has gained prominence, with several but not all Guideline groups recommending dose reductions for particular variant genotypes with the use of a common drug used in solid tumors – 5‐fluorouracil (5FU). However, there is increasing concern that dosing recommendations on genotypes are based on limited data sets and may lead to unnecessary underdosing and increased cancer mortality. In this Symposium, we presented our real‐world data of exposures of 5FU based on current dosing recommendations; we noted there was significant under‐dosing of 5FU in practice, in 61% of patients.^25^ Further there were significantly poor exposures in patients who were dihydropyrimidine dehydrogenase heterozygotes who would not metabolise 5FU sufficiently leading to toxicity; these patients may have reduced survival. The era of precision medicine is exciting, but the limitations of genetic testing, the lack of strong clinical benefit for most, and the need to understand phenotypes are increasingly evident.

### 1.5 Precision medicine for cystic fibrosis: Matching modulators to mutations. (D.N. Sheppard)

The monogenetic disease CF epitomizes both the success of precision medicine and its challenges through the intricacies of matching modulators to mutations. Since 2019, highly effective orally bioavailable modulators that rescue mutations in CFTR have transformed CF from an incurable life‐shortening disease to a treatable chronic condition for most, but not all people with CF.

CFTR is a highly polymorphic human gene with >2100 mutations, many affecting residues important for CFTR expression and/or function as an epithelial anion channel.^26^ Although most mutations are extremely rare, there is one notable exception: F508del, one copy of which is carried by approximately 90% of people with CF worldwide. To assist therapy development, mutations are classified by their mechanism of CFTR dysfunction: class I (defective protein production); class II (defective protein processing); class III (defective channel regulation); class IV (defective channel conduction); class V (reduced protein synthesis); and class VI (reduced protein stability). Few mutations cause CFTR dysfunction by a single mechanism (e.g., G551D, class III). The majority have multiple mechanisms of CFTR dysfunction (e.g., F508del, classes II‐III–VI). The latest iteration of the classification of CFTR mutations, a combinatorial scheme with 31 possible classes, is an important tool to match modulators to mutations for precision medicine in CF.^26^

Two types of modulators are currently licensed to treat CF. CFTR correctors overcome the misfolding and mis‐assembly of CFTR domains to permit the delivery of mutant CFTR protein to the plasma membrane, whereas CFTR potentiators enhance CFTR channel gating.^27^ Thus, CFTR correctors increase the number of channels available to transport anions, while CFTR potentiators augment the activity of individual channels.^27^ The combination of the correctors, elexacaftor, and tezacaftor, with the potentiator, ivacaftor (Trikfta^®^/Kaftrio; VX‐445‐VX‐661‐VX‐770; Vertex Pharmaceuticals, Boston, MA, USA) is a highly effective modulator therapy for people with CF heterozygous for F508del. Real‐world data demonstrate convincingly that elexacaftor‐tezacaftor‐ivacaftor has exceptional health benefits for people with CF.^28^

Based on the efficacy and safety of elexacaftor‐tezacaftor‐ivacaftor and undisclosed in vitro data demonstrating that the combination therapy restores channel function to different mutations expressed in heterologous cells, in 2020 the FDA further extended the use of elexacaftor‐tezacaftor‐ivacaftor to 177 additional CFTR mutations. Surprisingly, 74 of these additional mutations approved for treatment may not respond to the combination therapy.^29^, ^30^ Raraigh et al.^30^ designated four classes of non‐responding mutations: (i) non‐CF‐causing mutations; (ii) mutations of unknown significance; (iii) mutations that may affect splicing and (iv) mutations that occur as complex alleles with non‐modulator responsive mutations. To guide the further expansion of the elexacaftor‐tezacaftor‐ivacaftor label, Bihler et al.^31^ identified 292 rare CF‐causing mutations that respond to elexacaftor‐tezacaftor‐ivacaftor from a total of 655 tested in heterologous cells. Thus, while the predominance of the F508del mutation greatly assisted the development of precision medicine for CF, the scarcity of most CF‐causing mutations necessitates innovative drug development strategies, including the use of in vitro data, to optimize the matching of modulators to mutations.

## 2 SUMMARY

These expert presentations highlight key areas of progress and current challenges to the application of precision medicine across a larger array of diseases and targeted therapeutic interventions. The transformational promise of precision medicine is presently illustrated in the genomic‐based improvements in the management of some DDIs, targeted cancer therapies, and the treatment of orphan diseases like CF. Furthermore, these presentations also provide important drug discovery and translational research lessons for the development of disease biomarkers and novel drug therapies. For example, the emergence of CFTR modulator therapy for CF has fundamentally changed the disease trajectory for patients with the F508del mutation. This remarkable advance could not have occurred without effective collaborative partnerships between patient advocacy groups, research teams in academia and industry, and governmental regulatory authorities. In an era where the reliability and reproducibility of biomedical research have received much scrutiny, the precision medicine approaches presented in this symposium illustrate the translation of sound science into effective therapeutic interventions for patients, particularly in diseases like CF where patients with rare CFTR mutations render traditional randomized controlled trials unfeasible.

## AUTHOR CONTRIBUTIONS

All authors drafted, critically reviewed and approved the final version of the commentary.

## CONFLICT OF INTEREST STATEMENT

The authors declare no conflicts of interest.

## ETHICS STATEMENT

All the participants gave written informed consent and these are the trial registration data “Clinical Trials.gov, NCT04121195. Registered on 09 October 2019, https://clinicaltrials.gov/ct2/show/NCT04121195”

## Data Availability

Data available upon request
